# Surgical Management of Placenta Accreta Spectrum: A Five-Year Institutional Experience

**DOI:** 10.7759/cureus.94707

**Published:** 2025-10-16

**Authors:** Sudwita Sinha, Mukta Agarwal, Indira Prasad, Upasna Sinha, Muskan Rani

**Affiliations:** 1 Obstetrics and Gynaecology, All India Institute of Medical Sciences, Patna, Patna, IND; 2 Radiodiagnosis, All India Institute of Medical Sciences, Patna, Patna, IND

**Keywords:** antenatal diagnosis, caesarean hysterectomy, maternal morbidity, placenta accreta spectrum, surgical outcomes

## Abstract

Background: Surgical management of placenta accreta spectrum (PAS), particularly in emergency settings, poses significant risks. Although PAS is being encountered more frequently, there is still limited institutional data available to guide management. This study was aimed at evaluating the surgical outcomes in a tertiary care institution over five years and contributing to the growing body of evidence that guides the surgical management of PAS.

Methods: A retrospective review was conducted on 18 surgically managed PAS cases from April 2020 to March 2025. Demographics, intraoperative parameters, and maternal outcomes were analyzed. Comparative and regression analyses were performed.

Results: Mean maternal age was 30.6 years. Mean estimated blood loss (EBL)was 1822 ml (range 600-3600 ml), and mean operating time was 85.3 minutes (range 40-170 minutes). Emergency surgery was significantly associated with higher blood loss (p = 0.0002). Regression analysis revealed that emergency surgery was associated with more significant blood loss (β = 1,918 ml, p = 0.001).

Conclusion: Early antenatal diagnosis and planned surgeries are key to improving maternal outcomes. Multidisciplinary teams and preparedness are critical for optimal results. Our five-year institutional experience demonstrates that surgical management of PAS remains a high-risk intervention. Planned surgical approaches with antenatal diagnosis significantly improve outcomes by enabling resource readiness and effective team coordination. Emergency procedures, by contrast, are fraught with complications, further reinforcing the necessity for early risk identification and referral to equipped centers.

## Introduction

Placenta accreta spectrum (PAS) disorders represent a spectrum of abnormal placental implantation conditions, including placenta accreta (adherence to the myometrium), increta (invasion into the myometrium), and percreta (penetration through the uterine serosa and potentially into adjacent organs such as the bladder) [1]. These conditions are associated with significant maternal morbidity and mortality, primarily due to massive postpartum hemorrhage and shock, the need for hysterectomy, multiorgan dysfunction, disseminated intravascular coagulation, and, in severe cases, maternal death [2-6].

The rising global rates of cesarean delivery over the past three decades have been directly implicated in the increasing incidence of PAS. Current estimates place its occurrence between 0.12% and 0.31% of pregnancies, with the risk of PAS increasing exponentially with the number of previous cesarean sections, from a risk as low as 3% for the first cesarean section to as high as 67% for the fifth or subsequent cesarean sections [1,7]. Recent studies quote the mortality rate of PAS to be 7% [1]. Placenta previa, particularly in the context of a previous cesarean delivery, remains the most significant risk factor [7]. Other contributors include uterine surgeries such as myomectomy, dilation and curettage, manual removal of the placenta, hysterotomy, and the use of assisted reproductive technologies [1,7].

From a pathophysiological perspective, PAS is characterized by a failure of normal decidualization and excessive trophoblastic invasion into scarred uterine tissue [8]. Disruption in normal placental implantation is often linked to inadequate spiral artery remodeling, extracellular matrix defects, and immune dysregulation in the setting of a previously scarred endometrium [8]. Advances in molecular biology have highlighted the role of inflammatory cytokines and altered gene expression profiles in these aberrant processes [8].

The clinical management of PAS poses significant challenges and requires a high degree of surgical expertise, access to blood products, and intensive postoperative care [1,7]. The cornerstone of management for confirmed PAS remains cesarean hysterectomy without attempting placental removal, which the American College of Obstetricians and Gynecologists (ACOG) also recommends [3,4,5,7,9]. Scheduled preterm delivery, ideally between 34 and 36 weeks, is recommended to prevent emergency situations such as antepartum hemorrhage, premature labor, or fetal distress, which often result in poorer maternal and neonatal outcomes. However, conservative approaches such as leaving the placenta in situ, uterine artery ligation, or use of interventional radiology may be considered in carefully selected patients, especially those desiring future fertility [3,7,10,11]. Optimal management of these women remains a significant challenge, particularly in choosing between the two main strategies: cesarean hysterectomy and conservative management [12, 13]. In addition, the management of PAS may involve various adjunctive interventions to achieve hemostasis and minimize complications. These can be implemented preoperatively, such as ureteral stenting or intravascular balloon catheter placement; intra- or postoperatively, including uterine cavity packing or balloon tamponade, uterine compression sutures (B-Lynch, Hayman, or Cho square suturing technique), pelvic vessel ligation, abdominal aortic or internal iliac artery balloon occlusion, or prophylactic arterial embolization [6,7,12-22]. However, none of these approaches is currently supported by high-quality evidence specific to this condition, and their use has varied over time [16].

Prenatal diagnosis through active screening for PAS in patients with risk factors plays a crucial role in optimizing outcomes [7,9,12]. Ultrasound, supplemented by magnetic resonance imaging (MRI) in complex cases (such as posterior placenta), enables early detection, risk stratification, and timely referral to tertiary centers equipped with multidisciplinary teams experienced in PAS management [7,12,13,14,23]. Diagnostic features suggestive of PAS on ultrasound include retroplacental myometrial thinning (<1 mm), vascular lacunae, and retroplacental vascularization, the “riddled cervix” sign [7,12]. Scheduled preterm delivery, ideally between 34 and 36 weeks, is recommended to prevent emergency scenarios that often result in poorer outcomes [2,3,5,6,9,12,15]. Preventive strategies to reduce the incidence of PAS should include reducing the rate of Cesarean sections or using an endometrium-free closure technique [4].

Recent publications have reported innovations, such as the bladder filling technique, posterior surgical approach, bladder-first approach, double-row transfixation suture of the lower uterine segment, and the Soleymani and Collins (SAC) incision abdominal entry technique developed by the Oxford Placenta Accreta Team (OxPAT) to reduce complications such as bladder injury and blood loss and to ease retroperitoneal access during surgery [1,15,19,24-26]. The use of machine learning (ML) and radiomics has significantly improved the diagnosis and management of placenta accreta spectrum (PAS) [27]. Advanced imaging techniques, particularly MRI and ultrasound texture analysis, combined with machine learning (ML) algorithms, including deep learning, enable earlier and more accurate detection in high-risk pregnancies [27].

Despite its increasing frequency and clinical significance, PAS remains a relatively understudied condition due to its rarity and the absence of large randomized controlled trials. Consequently, much of the current guidance is based on retrospective studies and expert opinion, leading to variations in practice across institutions.

In this context, we conducted a retrospective review of PAS cases surgically managed over five years at a tertiary care center in India. The objective of this retrospective review was to evaluate the surgical outcomes of women with PAS managed at a tertiary care center over a five-year period. The primary outcomes assessed included intraoperative blood loss, need for hysterectomy, surgical complications, and postoperative morbidity such as intensive care unit (ICU) admission. The study also aimed to compare maternal and surgical outcomes between elective and emergency surgeries and between antenatally diagnosed and intraoperatively detected cases and to identify predictors of blood loss through regression analysis.

## Materials and methods

Study design and setting

This retrospective, single-center observational study was conducted at the Department of Obstetrics and Gynaecology, All India Institute of Medical Sciences (AIIMS) in Patna,a tertiary referral and teaching hospital in eastern India. The study was conducted over a period of five years, from April 2020 to March 2025.

Study population

The study included all pregnant women diagnosed with PAS, either preoperatively through imaging or intraoperatively during cesarean section, who underwent surgical management in a single dedicated obstetrics unit. Diagnosis of PAS was made preoperatively using ultrasonography, supplemented by MRI in cases with inconclusive ultrasound findings or posterior placentation, and confirmed intraoperatively when applicable. Patients were included regardless of the degree of PAS (accreta, increta, or percreta). Diagnosis of PAS was based on antenatal imaging findings and confirmed intraoperatively or histopathologically, where feasible. Ultrasound criteria included the presence of multiple placental lacunae, loss or thinning (<1 mm) of the retroplacental hypoechoic zone, turbulent lacunar blood flow on color Doppler, and abnormal uterovesical hypervascularity. MRI criteria included uterine bulging, dark intraplacental bands on T2-weighted images, and focal interruption of the myometrial wall. In cases where a hysterectomy was performed, histopathological examination of the uterine specimen was used to confirm the diagnosis and classify the depth of invasion (accreta, increta, or percreta).

Data collection

Data were extracted retrospectively from patient medical records, operative notes, imaging reports, and discharge summaries. The following variables were collected.

Maternal Demographics

Information on the patients' age, parity, obstetric history, and comorbidities was collected.

Obstetric and Surgical History

This included information on the number of previous cesarean sections, prior uterine surgeries (myomectomy, dilation and curettage, etc.)

Diagnosis

Timing of diagnosis (antenatal vs. intraoperative), gestational age at diagnosis, imaging findings (ultrasound/MRI), and placenta location were recorded.

Surgical Details

Gestational age at surgery, type of skin and uterine incision, operative time (from incision to closure), estimated blood loss (EBL), interventional procedures (e.g., arterial balloon occlusion, ureteric stenting), bladder or bowel injuries, and need for hysterectomy or conservative surgery were recorded. Adjunct procedures such as internal iliac artery ligation, ureteric stenting, or use of balloon occlusion were performed at the discretion of the operating team based on intraoperative blood loss, extent of placental invasion, and resource availability. The decision for adjunct procedures (such as internal iliac ligation, ureteric stenting, or balloon occlusion) was individualized based on intraoperative blood loss (>1500 ml), extent of placental invasion, and multidisciplinary team judgment. Internal iliac ligation was typically undertaken for uncontrolled hemorrhage exceeding 1500 ml or in cases of percreta where vascular invasion was evident. Conservative management was defined as surgical control of hemorrhage without performing hysterectomy, using techniques such as leaving the placenta in situ, uterine artery ligation, uterine compression sutures, or balloon tamponade.

Cesarean hysterectomy followed standardized steps, including midline laparotomy or Pfannenstiel incision (depending on exposure needs), delivery of the fetus via high transverse uterine incision, avoidance of placental removal, stepwise devascularization, and en bloc hysterectomy.

Postoperative Outcomes

This included ICU admission, ventilatory support, transfusion requirements, thrombotic or infectious complications, length of hospital stay, readmissions, and maternal mortality. Transfusion was guided by institutional massive transfusion protocols. Packed red blood cells were transfused when intraoperative hemoglobin was estimated below 8 g/dL or in cases of hemodynamic instability, irrespective of baseline levels. Fresh frozen plasma and platelets were administered as per the coagulation profile results or active bleeding.

Definitions

Scheduled Surgery

Elective surgery was scheduled for asymptomatic patients based on antenatal diagnosis of PAS at a planned time and carried out by an interdisciplinary team.

Emergency Surgery

This entailed urgent delivery performed due to antepartum hemorrhage, labor, or fetal distress at an unplanned time, before the scheduled date, or within the first six hours after hospital admission.

EBL

EBL was calculated based on suction canister volumes (excluding amniotic fluid), the number of soaked pads, and visual estimation by the surgical and anesthetic team.

Conservative management was defined as surgical control of hemorrhage without performing a hysterectomy, using techniques such as leaving the placenta in situ, uterine artery ligation, uterine compression sutures, or balloon tamponade.

Cesarean hysterectomy followed standardized steps, including midline laparotomy or Pfannenstiel incision (depending on exposure needs), delivery of the fetus via high transverse uterine incision, avoidance of placental removal, stepwise devascularization, and en bloc hysterectomy.

Ethical considerations

The study protocol was reviewed and approved by the Institutional Ethics Committee of AIIMS, Patna (approval no. AIIMS/Pat/IEC/2022/1026). Due to the retrospective nature of the study and the anonymized data presentation, informed consent was waived.

Statistical analysis

Data was entered and analyzed using IBM SPSS Statistics software, version 26.0 (IBM Corp., Armonk, NY). Continuous variables were tested for normality using the Shapiro-Wilk test. Continuous variables were presented as mean ± standard deviation (SD) if normally distributed, or median and interquartile range (IQR) if not. Categorical variables were summarized as frequencies and percentages. Comparative analyses were done using the chi-square test or Fisher’s exact test for categorical variables and the independent t-test or Mann-Whitney U test for continuous variables. Logistic regression was used to identify independent predictors of key binary outcomes. Linear regression was used for continuous outcomes. Variables included in regression models were selected based on clinical relevance and significant associations observed in univariate analysis (p < 0.1). Prior to model construction, multicollinearity was assessed using the variance inflation factor (VIF), and variables with VIF > 5 were excluded. Given the small sample size, bootstrapping with 1000 samples was used to validate regression coefficients and ensure model stability. Statistical significance was set at p < 0.05.

Strengths of the study methodology include comprehensive inclusion of all surgically managed PAS cases over five years, detailed intraoperative and postoperative data collection, and appropriate use of statistical techniques for small sample validation, thereby ensuring analytical robustness despite limited case numbers.

## Results

A total of 18 patients diagnosed with PAS were managed surgically over the study period in a single dedicated obstetrics unit. The mean age of the patients was 30.6 years. The average gravida was 3.0 ± 1.14, with a parity of 1.5 ± 0.86 and an abortion history of 0.5 ± 0.51. Patients had an average of 1.44 ± 0.70 prior cesarean sections. Placenta previa was present in 88.9% (16/18) of cases. Only one patient (5.6%) had a history of previous myomectomy. Elective surgeries were performed in six (33.3%) patients, while emergency surgeries were performed in 12 (66.7%) out of 18 patients. Antenatal and intraoperative diagnoses consisted of 50% each. The mean gestational age at diagnosis was 32 weeks. On classifying patients based on PAS severity, percreta was observed in eight (44.4%), accreta in six (33.3%), and increta in four (22.2%) out of 18 patients. The mean gestational age at diagnosis was 32.6 weeks, and at surgery it was 34.6 weeks.

Outcomes and complications

The Pfannenstiel incision was used in 72.2% of the surgeries, while the midline vertical incision was used in 27.8%. Conservative surgery was done in 50%, whereas the remaining 50% underwent hysterectomy. Internal iliac ligation was performed in 55.6% of cases. The mean EBL was 1822 ml (range: 600-3600 ml). The mean operating time is 85.3 minutes (range: 40-170 minutes). All patients (100%) received intraoperative blood transfusions. Postoperative blood transfusion was needed in 66.7% (12/18) of cases. ICU admission was required for 38.9% (7/18) of patients. Bladder injury occurred in 38.9% (7/18). Bowel injury and ureteric injury were each observed in 5.6% (1/18) of cases. Infections were reported in 11.1% (2/18) of patients. Disseminated intravascular coagulation (DIC) occurred in 22.2% (4/18) of the cases. Readmission within the postoperative period was required in 16.7% (3/18) of patients. There was one maternal death, yielding a mortality rate of 5.6%.

Emergency surgeries were associated with significantly higher blood loss compared to elective surgeries (p = 0.0002). Table 1 summarizes the comparison of key intraoperative variables between elective and emergency surgeries.

**Table 1:** Group Comparison: Elective vs. Emergency Surgery (Independent t-test)

Variable	t-statistic	p-value	Significance
Estimated blood loss	-4.98	0.0002	Significant
Operating time	-1.88	0.086	Not significant
Previous cesarean sections	-0.53	0.601	Not significant
Parity	-0.7	0.493	Not significant

Parity showed a borderline association with the timing of diagnosis (p = 0.054). Table 2 presents the comparison between antenatal and intraoperative diagnosis groups. Patients with antenatal diagnoses had significantly more previous cesarean sections (p = 0.041), indicating stronger screening among high-risk women.

**Table 2:** Group Comparison: Antenatal vs. Intraoperative Diagnosis (Independent t-test)

Variable	t-statistic	p-value	Significance
Estimated blood loss	-1.15	0.268	Not significant
Operating time	-0.36	0.722	Not significant
Previous cesarean sections	2.23	0.041	Significant
Parity	2.11	0.054	Borderline

There is a statistically significant association between PAS severity and internal iliac ligation (p < 0.001). Specifically, accreta cases rarely required ligation, and percreta cases almost always involved internal iliac ligation. Emergency surgery was independently associated with significantly increased blood loss (β = 1918 ml, p = 0.001) (Table 3). Linear regression analysis was performed to identify independent predictors of EBL. Emergency surgery was found to be the only significant predictor of increased blood loss (p = 0.001).

**Table 3:** Linear Regression: Predictors of Estimated Blood Loss (ml) (Multivariate Linear Regression Model)

Predictor	Coefficient (β)	p-value	95% CI for β	Interpretation
Previous cesarean sections	653.22	0.546	-1624, 2930	Not significant
Parity	-1098.81	0.226	-2965, 767	Not significant
Emergency surgery	1918.38	0.001	960, 2877	Significant
Antenatal diagnosis	738.42	0.142	-281, 1758	Not significant

## Discussion

Our findings highlight the high prevalence of placenta previa and multiple prior cesarean sections among PAS cases, underscoring the need for multidisciplinary surgical planning and readiness for complications such as DIC and urological injury. The observed rates of transfusion, ICU admission, and postoperative morbidity reflect the inherent complexity of PAS management and align with previously reported trends from tertiary referral centers.

The findings of our study also highlight the clinical and surgical challenges associated with managing PAS, particularly in emergencies. A key observation was that two-thirds of the surgeries were performed on an emergency basis, which correlated with significantly higher estimated blood loss and increased incidence of intraoperative and postoperative complications. This finding aligns with existing literature indicating that emergency surgical management of PAS is associated with significantly higher maternal morbidity and blood loss, largely due to limited preparedness, lack of multidisciplinary coordination, and delayed diagnosis [12,17,19,22].

Our data also indicate that antenatal diagnosis was associated with a more significant number of prior cesarean sections, suggesting that high-risk individuals with known obstetric histories are more likely to be screened. The predominance of placenta percreta in our series mirrors findings from other tertiary care studies, where referral bias leads to overrepresentation of severe forms of PAS [6,17,20]. This may be attributable to referral bias, where more severe cases are directed to centers equipped for complex surgical care. Moreover, our intraoperative transfusion rate of 100% highlights the massive hemorrhagic potential of PAS surgeries, emphasizing the need for advanced blood bank support and preoperative optimization.

Postoperative complications such as DIC, infections, and ICU admissions further demonstrate the systemic impact of PAS. Though the overall maternal mortality was relatively low (5.6%), the spectrum of morbidities necessitates a high index of suspicion and early multidisciplinary planning.

Importantly, regression analysis identified emergency surgery as a strong predictor of increased blood loss. This finding aligns with global data that stresses the importance of timely diagnosis and planned cesarean hysterectomy in a controlled environment. Our results advocate for strengthening antenatal surveillance protocols, particularly for women with prior uterine surgeries, and ensuring the availability of multidisciplinary surgical teams, anesthesiologists, interventional radiologists, and critical care support.

Our results underscore the importance of early antenatal diagnosis, multidisciplinary surgical planning, and preparedness with blood products and intensive care support. These findings are consistent with large multicenter analyses emphasizing the impact of scheduled cesarean hysterectomy on maternal safety [2,5,12,15]. The high rate of bladder injury (38.9%) and need for transfusion in our series reflect the complex surgical anatomy in percreta cases, as similarly reported by Kandemir et al. [18] and Abouda et al. [19]. The data reinforce the recommendation that PAS cases be managed at high-volume tertiary centers with experienced obstetric, anesthetic, and urological teams.

In summary, our five-year institutional experience demonstrates that surgical management of PAS remains a high-risk intervention. Planned surgical approaches with antenatal diagnosis significantly improve outcomes by enabling resource readiness and effective team coordination. Emergency procedures, by contrast, are fraught with complications, further reinforcing the necessity for early risk identification and referral to equipped centers.

The strengths of this study lie in its comprehensive inclusion of all surgically managed PAS cases over five years, minimizing selection bias and reflecting real-world clinical practice in a tertiary referral center. Detailed intraoperative and postoperative parameters were analyzed, allowing identification of specific predictors of adverse outcomes such as blood loss. The use of regression analysis and bootstrapping enhanced the reliability of statistical inference despite a small sample size. Moreover, the study adds valuable data from a low- and middle-income country (LMIC) setting, where resource constraints significantly influence maternal outcomes, an aspect underrepresented in the global literature.

This study has several limitations. The small sample size (n=18) limits statistical power and generalizability, and findings should be interpreted as exploratory rather than confirmatory. As a single-center study conducted at a tertiary referral hospital, the data likely overrepresent severe PAS cases due to referral bias, potentially leading to higher observed morbidity and transfusion rates. The absence of a control or conservatively managed comparison group prevents assessment of relative effectiveness across management strategies. Additionally, variations in imaging interpretation, surgical techniques, and the use of adjunct procedures reflect real-world practice but introduce non-standardization that could influence outcomes. Finally, only short-term surgical outcomes were analyzed; long-term maternal health, reproductive potential, and psychological impact were not assessed. Despite these limitations, the study provides valuable insights into PAS management in a low-resource tertiary care setting.

## Conclusions

In this single-center retrospective cohort of 18 surgically managed PAS cases, emergency surgical management was associated with markedly higher intraoperative blood loss (p = 0.0002) and increased postoperative morbidity, including greater transfusion requirements and intensive care admissions. Planned or elective surgeries, supported by antenatal diagnosis and multidisciplinary preparedness, were associated with comparatively better maternal outcomes in this cohort.

These findings suggest that early antenatal detection and scheduled surgical intervention may improve outcomes by allowing adequate preparation and coordinated team management; however, this represents an association rather than a causal relationship.

The conclusions must be interpreted in light of the study’s limitations, including its retrospective design, small sample size, single-institution setting, and potential referral bias toward severe cases. Despite these limitations, the study provides valuable insight into real-world surgical outcomes and reinforces the importance of early risk identification and management planning for PAS, particularly in resource-constrained settings.
